# Efficient deactivation of aerosolized pathogens using a dielectric barrier discharge based cold-plasma detergent in environment device for good indoor air quality

**DOI:** 10.1038/s41598-023-37014-2

**Published:** 2023-06-25

**Authors:** Ramavtar Jangra, Kiran Ahlawat, Ambesh Dixit, Ram Prakash

**Affiliations:** Department of Physics, Indian Institute of Technology Jodhpur, Jodhpur, Rajasthan 342037 India

**Keywords:** Plasma physics, Catalyst synthesis, Environmental sciences

## Abstract

Air pollution is one of the top 5 risks causing chronic diseases according to WHO and airborne transmitted pathogens infection is a huge challenge in the current era. Long living pathogens and small size aerosols are not effectively dealt with by the available indoor air purifiers. In this work, a dielectric barrier discharge (DBD) based portable cold-plasma detergent in environment device is reported and its disinfection efficiency has been analyzed in the indoor environment of sizes up to 3 × 2.4 × 2.4 m^3^. The deactivation efficiency of total microbial counts (TMCs) and total fungal counts (TFCs) is found to be more than 99% in 90 min of continuous operation of the device at the optimized parameters. The complete inactivation of MS2 phage and *Escherichia coli* bacteria with more than 5 log reduction (99.999%) has also been achieved in 30 min and 90 min of operation of the device in an enclosed environment. The device is able to produce negative ions predominantly dominated by natural plasma detergent along with positive ions in the environment similar to mother nature. The device comprises a coaxial DBD geometry plasma source with a specially designed wire mesh electrode of mild steel with a thickness of 1 mm. The need for feed gas, pellets and/or differential pressure has been eliminated from the DBD discharge source for efficient air purification. The existence of negative ions for more than 25 s on average is the key advantage, which can also deactivate long living pathogens and small size aerosols.

## Introduction

In the current era, airborne transmitted pathogen infection is causing diseases of significant morbidity and mortality^1^. Almost every year there appears a new bacteria or virus of influenza nature that creates an epidemic or pandemic of diseases^2^. Besides human-to-human transmission, in the highly crowded and indoor enclosed environments such as healthcare facilities, schools, colleges, universities, large shopping malls, commercial buildings, and public buildings, indoor pathogens shed from humans may further transmit and disperse through heating, ventilation, and air conditioning (HVAC) systems, and may lead to cross-infections. This fear created a lockdown across the world and the infections due to the SARS-CoV-2 virus affected work productivity hugely^3^. In general, people spend 70–90% of their time in indoor environments^4^. Indoor air quality (IAQ) is quite important for personal health safety, which is in general 2–5 times or even more polluted than outdoor air^5^. Many researchers have worked on different methods to reduce the risk of microbial infections in indoor spaces and for improving IAQ^6–11^.

One of the known techniques is chemical disinfection, which makes use of ethanol (C_2_H_5_OH), hydrogen peroxide (H_2_O_2_), or a disinfectant^12^. Microorganisms can be eliminated as soon as they come into direct contact with these chemicals. However, it is challenging to decontaminate a big volume using this method. Another method of disinfection uses gases like ethylene oxide (C_2_H_4_O) or ozone (O_3_) to prevent microbial infection^13,14^. With these methods, the sterilization is consistent across the treated volume, but it is essential to isolate the area and set up a suitable exhaust ventilation system. High-efficiency particulate air (HEPA) filters are also used for removing dust particles and airborne microorganisms from indoor environments^15,16^. However, HEPA filters do not deactivate microorganisms, are unable to filter small size aerosols and also can cause pressure drops in air conditioning systems. Researchers have then moved on to ultraviolet (UV) and UV-based photocatalysis methods, which are quite promising for microbial reduction and indoor air purification^17,18^. Nevertheless, the UV-based approach requires a long sterilization time^19^, is not effective when the concentration of pollutants is lower^20^, and also UV light handling in public places is quite challenging^21^. Therefore, engineering control strategies for innovative solutions for efficient disinfection of airborne pathogens for broad use at a lower cost is the need of the hour.

A non-thermal plasma (NTP) technology has emerged in recent times, which has been studied largely for sterilization and indoor air purification^22–24^. High-energy electrons (1–10 eV) are produced in NTP, and the background gases remain near room temperature, which is also referred to as a cold plasma^25^. The main factors influencing the antimicrobial effects of NTP are localized electric fields, reactive oxygen species (ROS), and charged particles (positive and negative ions)^26^. In line with this, a 1.5 log reduction (97%) in culturable *E. coli* was achieved by a DBD NTP rector with a very high airflow rate (25 L/s) and a very short duration of plasma exposure^27^*.* The ozone concentration produced by the reactor was around 28 ppm, which is very high as per international health regulatory organizations’ standards^28^. The permissible level of ozone according to World Health Organization (WHO) is 0.05 ppm for 8 h exposure, according to Occupational Safety and Health Administration (OSHA) is 0.1 ppm for 8 h per day and 5 day per week, and according to National Institute for Occupational Safety and Health (NIOSH) is ≤ 0.1 ppm not be exceeded at any time. These data determined that the permissible level of ozone is around 0.1 ppm for 8 h exposure at workplaces^29^. Another study showed > 95% inactivation of bacteria and around 85–98% for fungal species with a DBD source^30^. They created plasma at a voltage and frequency of 14 kV and 10 kHz, respectively, with an airflow rate of 28.3 L/min.

A study carried by Nishikawa et al.^31^ has shown the effect of negative and positive charged ions generated by DBD NTP plasma at ambient pressure on *Klebsiella coli*. They used an alternating current (AC) power source to produce discharge and achieved a maximum of 82% deactivation efficiency by operating the device for 60 min. Wu et al.^32^ showed that negative air ions produced by air ionizers can be used to decompose different volatile organic compounds (VOCs). They addressed that the reaction between negative air ions and the VOCs is prolonged and complicated. Therefore, the plasma ionizers that can produce only negative air ions are not much effective in air purification applications. In another study, it was argued that negative air ions can be used for indoor air cleaning particularly for particulate matter (PM) concentration reduction^33^. They concluded that no data have shown the harmful effect of negative air ions on humans/animals from the available literature if these are produced in controlled concentration. In general, NTP produces harmful by-products, particularly ozone, potentially more detrimental to human health than the treated pollutants^34^.

The factor influencing the antimicrobial effects of NTP is the negative air ions^22^. Hydroxyl radicals ($\cdot {\text{OH}}$) (or their charged negative ions) are far more effective in the air than other disinfectants in oxidizing and removing pollutants^35^. They are several times more germicidal and fungicidal than other disinfectants (like alkali, chlorine, alkali-alcohol-amine, etc.), making them a promising candidate for destroying harmful bacteria and viruses from indoor environments^36^. Compared to other chemical disinfectants, plasma produced $\cdot {\text{OH}}$ (and negative ions) can have various advantages: (1) Due to their high oxidation potential (2.8 V), they can eliminate any pathogenic microorganisms in low fatal doses; this property is termed as the absence of selectivity^37^. (2) The reaction rate of $\cdot {\text{OH}}$ is several times greater than the other oxidants such as ozone, chlorine, etc.^38^ (3) they are considered to be natural detergents or “eco-oxidants”, as $\cdot {\text{OH}}$ naturally decomposes into water (H_2_O) and oxygen (O_2_) without leaving behind any residual oxidants once their biological reactions have finished^39^. Consequently, Nobel prize winner Paul Crutzen coined the phrase ‘Detergent of the Atmosphere’ for $\cdot {\text{OH}}$ and OH negative ions^40^. Martinez et al.^41^ showed the different scenarios of how $\cdot {\text{OH}}$ can react with different pathogenic microorganisms and VOCs. They found the efficacy of virus elimination, which was around 92–99% depending on humidity conditions. In another study, the synergetic effect of $\cdot {\text{OH}}$ and plasma-generated ozone further led to a record 97% in-flight inactivation of aerosolized *E. coli*^42^.

There is a range of methodologies tested to generate $\cdot {\text{OH}}$ using UV-light and/or plasmas^43,44^. The UV-light has constraints because the energy of the electron–hole pair is limited and the generated $\cdot {\text{OH}}$ quenches well before (i.e., ~ 0.1 s)^45^. Evidence of destroying viruses using non-thermal non-equilibrium (cold) plasma without the use of UV has been reported very recently^46^*.* They discussed the NTP exposure on viral aerosol particles in an airstream. The cold plasma was produced in a packed-bed DBD reactor. More than 2.3 log reduction of infectious virus was achieved throughout the reactor at 30 kV voltage and airflow at the rate of 170 standard litres per minute. Enhancing the airflow rate from 170 to 330 L per minute had no discernible effect on virus inactivation. In the packed-bed reactor, the electron-impact reactions serve as the main plasma chemistry for the air pollutant decomposition^47^. Plasma discharges which can produce $\cdot {\text{OH}}$ have also been tried^48,49^. They are either costlier or consume high power (like, sliding arc, microwave generated cold plasmas, fast pulsed high-pressure discharges, etc.).

In this paper, we present a cold-plasma detergent in environment device which produces simultaneously positive and negative ions along with $\cdot {\text{OH}}$ for high efficiency eradication of aerosolized pathogenic microorganisms in the enclosed indoor environments. The device is based on the concept of surface dielectric barrier discharge (SDBD) plasma in combination with TiO_2_ metal oxide nanoparticles catalyst. The developed process for the production of positive and negative ions, and plasma generated $\cdot {\text{OH}}$ is quite superior because these are produced by energetic electrons generated in the coaxial DBD geometry by cold plasma. Otherwise, these are produced separately in most cases, for example, $\cdot {\text{OH}}$ are in general produced by conventional metal oxide nano-catalysts irradiated by UV rays in a well-known photocatalytic oxidation process^18^. In our case, due to high electrostatic fields generated with random strength in the developed device, we are able to generate $\cdot {\text{OH}}$ as well as positive and negative ions similar to mother nature, simultaneously. Furthermore, we are able to sustain negative air ions on an average for more than 25 s, which would be quite useful to deactivate long living pathogens.

In this type of plasma discharge, the high-energy particles transfer their energy by collision with the metal oxide coated surfaces, and the pulse shape as well as the electrode geometry provide the control on the concentration of the active ions in the environment. Besides these, the developed device is easy to construct, cost-effective, and lightweight. The device disinfection efficiency has been analyzed for TMCs and TFCs and also on aerosolized *E. coli* bacteria and MS2 phage in an enclosed indoor environment. The obtained results are discussed in terms of the generated cold-plasma detergent in the environment and their effectiveness.

## Experimental setup

### Device design

**Figure 1:**
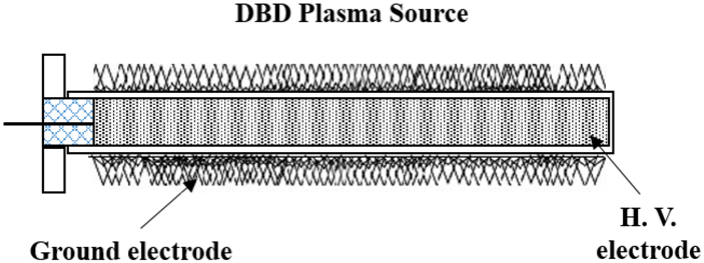
Schematic view of the coaxial DBD device.

The device comprises a coaxial DBD arrangement as shown in Fig. 1. An aluminum rod of length 95 mm is acting as a central high voltage (or powered) electrode. The grounded electrode is made of wire mesh of mild steel having a thickness of 1 mm. A cylindrical glass tube with an outer diameter of 16 mm, a glass thickness of 1.85 mm and a length of 100 mm is used as a dielectric barrier between the powered and grounded electrode. The aluminum rod is shielded hermetically into the glass tube, and the wire mesh is tightly fitted on the outer electrode. Polytetrafluoroethylene (PTFE) structure of length 15 mm is used as an end cap for insulation purposes. Titanium dioxide (TiO_2_) nanoparticles catalyst is coated on the outer grounded electrode via a dip-coating process to attain a strong coating. The TiO_2_ nanoparticles are prepared using the sol–gel method and the process is described in our earlier work^21^. First of all, the designed external electrode structure was subjected to the cleaning process and surface preparation under basic conditions. This cleaning exercise helped in removing any residual organic and other surface impurities for better adhesion of TiO_2_ nanoparticles on the surface of the used external electrode. Then using the aforementioned process, a uniform coating of thickness ~ 0.9–1.1 μm has been achieved in order to sustain electric fields of random strengths.

The plasma discharge is produced at atmospheric pressure, and ambient air is used as the gas carrier. A variable bipolar pulsed power supply (1–6 kV, 5–40 kHz PRF, 1 A, and 2 µsec pulse width) is used to produce surface dielectric barrier discharge. In this geometry, random local electric field generation is possible through the surface discharge, which is otherwise produced in volume dielectric discharge with much more complexities^50^.

### Characterization of TiO_2_ nanoparticles catalyst

**Figure 2:**
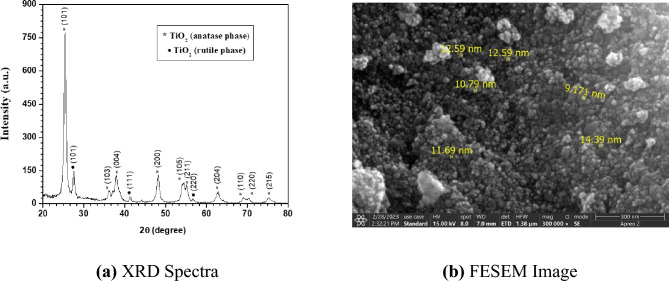
Characterization of the TiO_2_ nanoparticles synthesized by sol–gel method and annealed at 450 °C for 3 h.

The lab-synthesized powder consists of TiO_2_ nanoparticles, both in the anatase and rutile phases, in which anatase is the predominant phase. Figure 2a depicts the X-ray diffraction (XRD) pattern of the TiO_2_ nanopowder (annealed at 450 °C for 3 h) that revealed various strong diffraction peaks at 2θ values of 25.43° (101), 27.59° (101), 36.13° (103), 38.01° (004), 41.43° (111), 48.47° (200), 54.29° (105) 55.11° (211), 56.71° (220), 63.04° (24), 68.89° (110), 70.43° (220) and 75.31° (215) matching well with the TiO_2_ anatase and rutile phase. The average crystalline size of the TiO_2_ nanoparticles is estimated by Debye–Scherrer’s relationship^51^, and it is found to be ~ 17 ± 2 nm, confirming the nanoparticle formation of TiO_2_. These diffraction data are consistent with ICDD PDF 03-065-5714 for anatase TiO_2_ and ICDD PDF 03–065-1118 for rutile TiO_2_. To see the grain size and uniform distribution of prepared TiO_2_ nanoparticles, field emission scanning electron microscopy (FESEM) is used. Figure 2b shows the high magnification images of a synthesized TiO_2_ powder sample coated on the ground electrode. It can be noticed from the FESEM image that the average grain size of TiO_2_ nanoparticles is around 12–15 nm, which is compatible with crystallite sizes estimated from the XRD data. The porosity network can also be observed from the FESEM image, which is better for releasing reactive ionic species from the interior nanoparticles as well.

### Electrical characterization and air quality analysis

**Figure 3:**
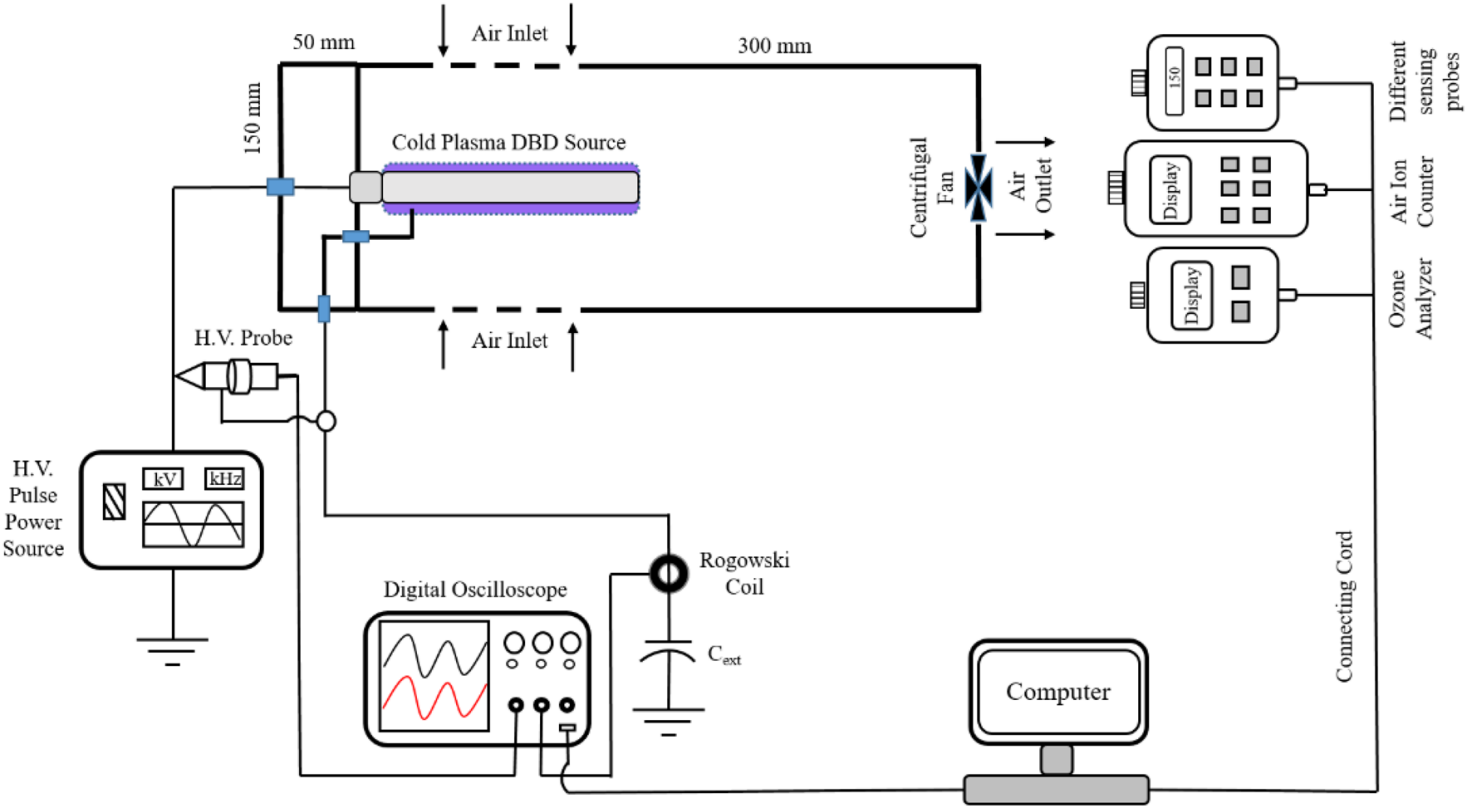
Schematic view of the experimental setup.

For electrical characterization and air quality analysis, a setup as shown in Fig. 3 is used. The applied voltage is measured by a high voltage probe (P6015A, Tektronix), and the discharge current is measured by a fast response current transformer (Pearson 110, Tektronix). A four-channel digital oscilloscope (MDO 4014-3, Tektronix) is used to visualize the discharge current and applied voltage waveforms. The negative and positive air ions are continuously generated during the plasma discharge, and the same is measured by an air ion counter (AIC2, AlphaLab, USA). A portable ozone monitor (ATS-101M, Applied Techno Systems), with a precision of 1 ppb, has been mounted separately with the experimental setup to measure the real-time concentration of ozone produced. Indoor air quality probes (IQ-610, GrayWolf Sensing Solutions, Shelton, CT USA) and toxic gas probes (TG-501, GrayWolf Sensing Solutions, Shelton, CT USA) are also used to measure the different parameters for indoor quality monitoring.

### Test setup for microbial analysis

The air purifying unit used for disinfection studies is shown in Fig. 3. The developed plasma device is placed inside a rectangular box made of stainless steel (SS) for detailed analysis. The dimensions of the SS box are 350 mm (L) × 150 mm (W) × 150 mm (H). For continuous air circulation, four holes (diameter of 20 mm) are made in the box as shown in this Figure, and a centrifugal fan is placed at the end to spread generated ions in the indoor environment. The used centrifugal fan delivers air with a flow rate of 225 m^3^/h.

For the assessment of microbial inactivation, the developed air purifying unit is tested in a room size measuring 3 × 2.4 × 2.4 m^3^ for TMCs and TFCs with and without ventilation by using an air sampler (HiMedia Laboratories, India). In TMCs and TFCs measurements, for the without ventilation case, a room of size 3 × 2.4 × 2.4 m^3^ was prepared. There was a negligible leak in the room. It was a special arrangement having two sliding glass windows in a space size of 0.6 × 0.6 m^2^ having centrally opening doors in a gap of 0.6 m at one side of the wall of this room. Rest it was air tight. One glass window opens inside the room and the other opens outside in the gallery. The air sampler was placed in the space between the glass windows, and the device was operated in the room continuously for 90 min. The air sampler was controlled remotely from the outside of the room and samples were collected manually with least exposure to the outside environment. For the ventilation case, another office room of size 3 × 2.4 × 2.4 m^3^ is used with no specific air tightness. The leaks were solely from the entrance gate and one window of size of 0.9 × 0.9 m^2^ that was closed but not airtight. Also, the room door was opened after every 15 min for sample collection. The device was kept in the middle of the room, and the air sampler was placed in one corner of the room. This testing was done to estimate the real-life scenario of TMCs and TFCs degradation efficiency by the developed device. The exact ventilation rate could not be measured.

For measuring the TMCs and TFCs, the Nutrient Agar (NA) plates for TMCs analysis and Potato Dextrose Agar (PDA) for TFCs analysis were used. The plates were prepared separately in the microbiological laboratory and kept in a suitable incubator for 24 h to check contamination. TMCs, as counts measure on NA, shows the total number of microorganisms present in a sample. Similarly, TFCs, as counts measured on PDA, shows the number of fungal present in a sample that has allowed the identification of viable yeast and mould species present. The air sampler could operate at numerous airflow sampling rates according to the desired test settings. The volume of air for sampling was 1000L. The air sampler was placed in the test room and air sampling has been carried out with the sampler before starting the device. After each experiment, the rooms used for testing were sufficiently equilibrated before next set. The control data were taken for each set of experiment and results were plotted accordingly. For the disinfection analysis, the following steps are followed;

1. The NA and PDA plates were prepared and placed in the air sampler for the cultivation of a wide variety of nonfastidious microorganisms from the room atmosphere at a fixed sampling rate of 1000L.
2. For every test sequence, background TMCs and TFCs were sampled before the subject prototype operation.
3. Samples were collected at different exposure times, i.e., 5, 10, 15, 20, 30, 45, 60, 90, and 120 min keeping the same test conditions.
4. The treated NA and PDA plates were kept for 48 h of incubation at 37 °C. After completion of the incubation period, colony-forming units (CFUs) are quantified using the conventional plating process for each exposure time.
5. Total CFUs reduction was estimated from the difference between the initial concentration of CFUs before operating the unit and the final concentration of CFUs after plasma exposure at different time intervals.

In another assessment for the disinfection efficiency, the developed air purifying unit is tested in a pre-cleaned closed test chamber (0.6 × 0.6 × 0.6 m^3^) along with an aerosol capture device, temperature and humidity meters, and having sampling and injection ports on one/opposite sides of the chamber, for the following two microorganisms, i.e., *Escherichia coli* gram-negative rods (ATCC Reference No. 8739) and MS2 phage single-stranded RNA virus (ATCC Reference No. 15597 B1) put deliberately at the organism control level. The host for the MS2 bacteriophage was *Escherichia coli*. All the test organisms are aerosolized to charge the test area with the desired test organisms using an externally operated nebulizer. The concentrations of *E. coli* and MS2 phage used for aerosolization in the nebulizer were 6 × 10^9^ CFU or PFU/ml. The total volume injected was 5 ml over a period of 5 min. The aerosols were allowed to stabilize for 1 min and after that aerosolization was stopped, which was kept same for testing and control. The device is operated for different time intervals, intermittent one after another. The charged air was captured after active running of device using AGI impinger and multi-stage active air sampler for enumeration of bacteria and viral survival on appropriate growth medium (with host in case of virus enumeration) for cultivation. After each run, the test chamber is disinfected using a fogger and disinfectant solution (SurOxyl). Throughout the analysis, the internal chamber condition is maintained at 25 °C and relative humidity < 60%.

Initially, *E. coli* bacteria and MS2 phage were grown in an appropriate growth medium. *E. coli* cultures were inoculated in TSB broth and were incubated for 12–16 h at 37 °C. After achieving the required optical density (approx. 0.8 at 560 nm), bacterial suspension cells were prepared in PBS from TSB broth. The prepared bacterial suspensions are used for aerosolization in the chamber. Similarly, phage plates are quantified in plaque-forming units (PFUs) for the virus, and a culture with an appropriate host cell is used in the experiment. After the plasma source operation in the test chamber, the sample plates are incubated at 37 °C for 72 h. The natural decay of *E. coli* and MS2 phage microorganisms has been adjusted in the survival data reported here.

## Results and discussion

**Figure 4:**
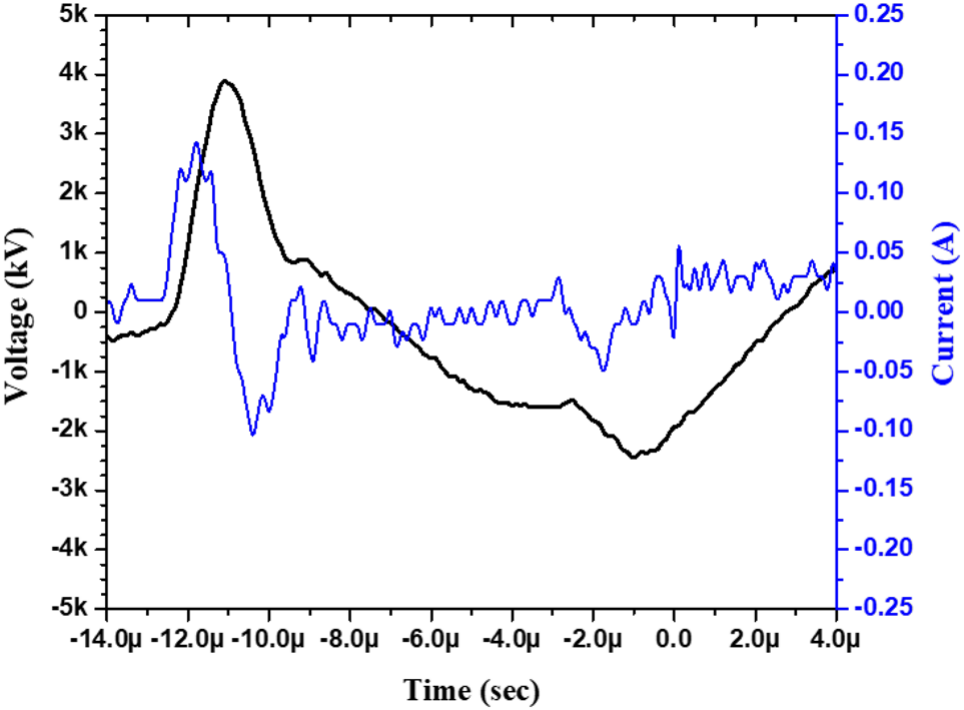
Typical V–I characteristics of the coaxial DBD source at 4 kV/20 kHz.

A typical V–I characteristic of the above described coaxial DBD source is shown in Fig. 4. The electric power dissipated into the plasma is calculated from the waveforms of applied voltage (U_t_) and the discharge current (i_t_) using the following relationship.

1 $$E_{p} = \mathop \smallint \limits_{t} U_{t} \times i_{t} dt$$

2 $$P = E_{p} \times f$$

where t is the period of bipolar pulsed voltage, E_p_ is the energy consumed per pulse (Joule), f is the pulse repetition frequency (Hz), and P is the discharge power (W).

The energy consumed per pulse has been estimated by integrating power over the pulse duration. This gives a pulse energy of 1.65 × 10^−4^ Joule at the applied frequency of 20 kHz with an applied pulse voltage of 4 kV (peak) and for a pulse width of 2 μsec. This results in 3.31 W average electrical power consumption in a coaxial DBD device of length 100 mm. The power consumed per unit arc length by this device is ~ 0.33 W/cm. From the V–I curve, the electron density can be estimated using the following relationship,

3 $${n}_{e }= \frac{J}{e{\mu }_{e}E}$$

where J is the current density, e is the electronic charge, and µ_e_ is the electron mobility, i.e., 552 cm^2^/V.s in the case of nitrogen^52^. The electron density turns out to be ~ 1 × 10^9^ particles/cm^3^ at the aforesaid operational parameters.

It is to be emphasized that when electron density is low, a rather simple ‘Corona’ equilibrium model, is in general used to understand the discharge plasma spectroscopically^53^. In the corona model, it happens that all upward transitions are collisional and all downward transitions are radiative. In fact, in a low-density plasma case (n_e_ ≤ 10^10^ cm^−3^), the probability for spontaneous decay of an excited atom is much higher than that of any collisional depopulation process. Hence it can be easily assumed that,

(i) All upward transitions are collisional
(ii) All downward transitions are radiative

This can only happen when transitions are mostly occurring in the excited states but near to the ground state, which implies that the spectrum at low electron densities mostly lies in the visible region^54^. Further, these assumptions are also valid even for n_e_ ≥ 10^10^ cm^−3^ but the transitions need to happen very near to the ground state. Such transition produces spectrum in the extreme ultraviolet (EUV) and/or vacuum ultraviolet (VUV) region, which is in one proof that there is almost nil UV generation during the discharge. A test done with a calibrated UV-C radiometer (UV Sensor—HS15-U and UV Monitor—HM-2) has shown zero UV-C generation during the discharge. Ozone concentration has also been measured around the air purifying unit using a portable ozone gas detector, which is found to be 24 ppb at the aforementioned operational parameters, which is within the permissible exposure limit (nil) as per the Centre for Disease Control (CDC) guidelines^55^.

**Figure 5:**
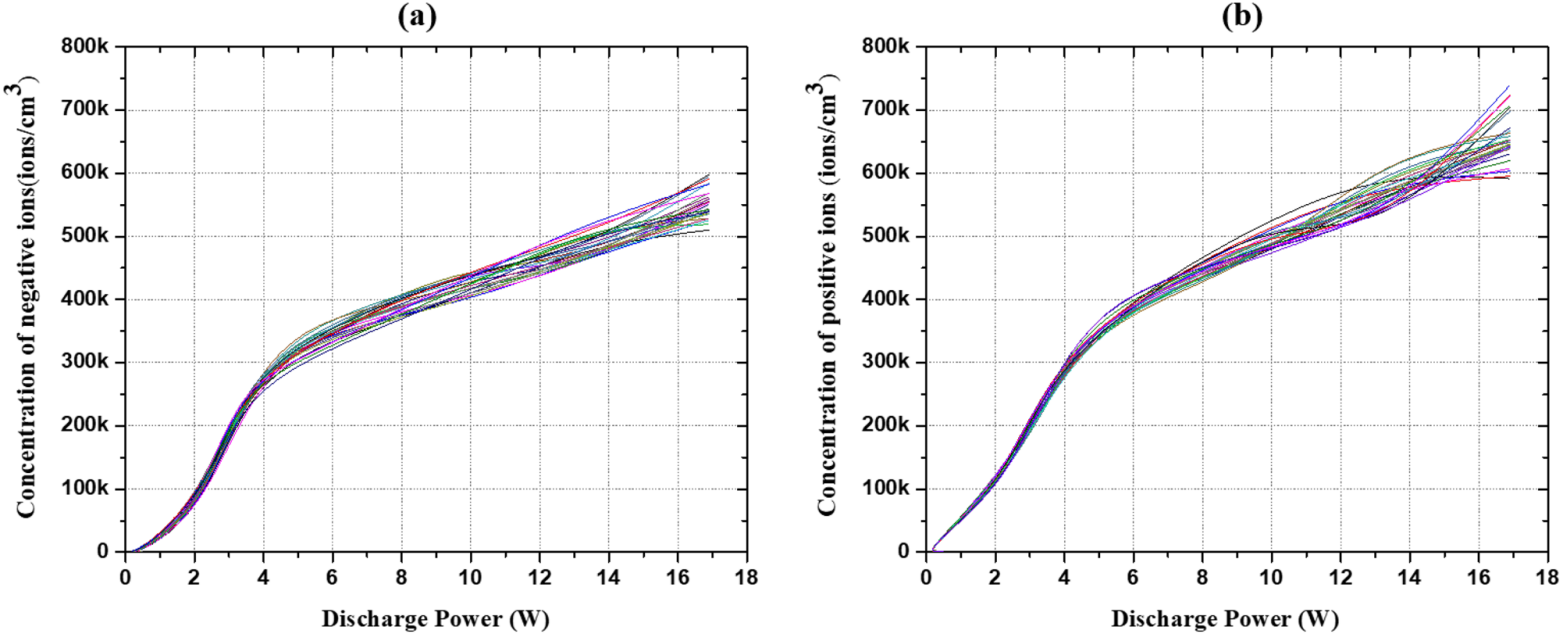
The concentrations of (**a**) negative and (**b**) positive air ions at different discharge powers at ambient conditions, monitored for 30 days.

Figure 5a,b depict the negative and positive ion concentrations measured by the air ion counter (AIC) from the device at the outlet of the air purifying unit for different discharge powers in an enclosed environment measuring size 3 × 2.4 × 2.4 m^3^. Positive and negative ions are generated by the molecules present in the air during their ionization by the discharge plasma produced at the contact points of the outer electrode. It is clearly seen that the densities of both negatively and positively charged ions enhance upon increasing the discharge power. The concentration of positive ions at the same discharge power is somewhat higher than the concentration of negative ions at ambient conditions. The measured value of negative and positive ions density is 2.30 × 10^5^ ions/cm^3^ and 2.42 × 10^5^ ions/cm^3^, respectively at the discharge power of 3.31 W. The concentrations of negative and positive ions produced from the device are such that they can yield local fields (> 10^6^ V/m) in the indoor environment similar to the bond energy of the chemical bonds of the harmful pathogens in the environment at their scales for faster deactivation.

We measured these positive and negative air ion concentrations for 30 days in the same indoor environment, and over that time the concentration approximately remained constant, indicating that there has been no deterioration in the electrodes and TiO_2_ coating. Furthermore, the local fields are such that the excess negative and positive ion concentrations are neutralized in air, which otherwise can deposit on to the surfaces—a major disadvantage of presently used single polarity ion-based air purifiers for dust capturing in the indoor environment. The negative ions obtained are a mix of hydroxyl ions produced by activating the TiO_2_ catalyst in the surface DBD discharge. Assadi et al.^56^ reported that the UV light generated via a surface DBD plasma was too weak to activate the TiO_2_ catalyst. Wallis et al.^57^ suggested that the impact of plasma-generated electrons on the surface of TiO_2_ can produce electron–hole pairs similar to photon absorption since the energy of these electrons, i.e., 3–4 eV are identical to the photons.

4 $${\text{TiO}}_{2} + e^{ - } \left( { > 3.2\;{\text{eV}}} \right) \to e^{ - } + h^{ + }$$

The recombination time of these electron–hole pairs is 2–3 times faster than the charge separation time. Since we are able to generate electric fields of random strengths, the recombination time of these electron–hole pairs would have reduced. This is well supported by Mizuno et al.^58^, they explained that the electric fields produced by the interactions of discharge plasma and metal oxide photocatalyst significantly shorten the time required for recombining electron–hole pairs. Probably electrons and holes produced by the highly energetic electrons would have been transported in the opposite direction due to the action of these randomly created electric fields and that can minimize the probability of their recombination. This can obviously lead to increased negative ion sustenance time.

**Figure 6:**
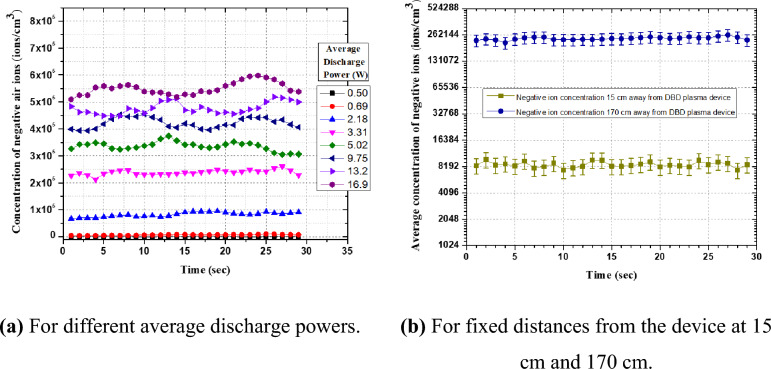
The recorded average concentration of negative air ions with their sustenance time.

Figure 6a shows the concentrations of the measured negative air ions and their sustenance time for different discharge operating parameters. We measured the negative ions concentration produced by the device at 15 cm from the source. The used AIC sucks the air at a calibrated rate and, in general, the display shows the ion count after 2 s that is continuously displayed. By using an analog output jack, the long-term automated monitoring of ion counts, i.e., up to 30 s is possible. Accordingly, the measured ion densities at different operating parameters have been traced for ~ 28 s. Since the average densities measured during 28 s is much higher than the original room ion concentration which was ~ 200 particles/cm^3^ in the closed test environment chosen in the study, it clearly shows that the average sustenance time of ions is more than 25 s. Accordingly, the average measurement of these ions has been used to show the concentrations of the plasma detergent ions in the environment. Figure 6b shows the average negative ion concentration at different distances from the source. The values of negative ion concentrations decreased with distance due to their expansion in the enclosed environment. Still, the average sustenance time of negative ions is the same.

The generated electron–hole pairs can lead to generate cold-plasma detergent (hydroxyl radicals and OH negative ions) in the environment by the following reactions:

5 $$h^{ + } + {\text{H}}_{2} {\text{O}} \to {\cdot\text{OH}} + {\text{H}}^{ + }$$

6 $$h^{ + } + {\text{OH}}^{ - } \to \cdot {\text{OH}}$$

7 $$e^{ - } + {\text{O}}_{2} \to \cdot {\text{O}}_{2}^{ - } + {\text{H}}^{ + } \to {\text{HO}}_{2} \cdot$$

8 $$2{\text{HO}}_{2} \cdot \to {\text{O}}_{2} + {\text{H}}_{2} {\text{O}}_{2}$$

9 $${\text{H}}_{2} {\text{O}}_{2} + \cdot {\text{O}}_{2}^{ - } \to \cdot {\text{OH }} + {\text{OH}}^{ - } + {\text{O}}_{2}$$

**Figure 7:**
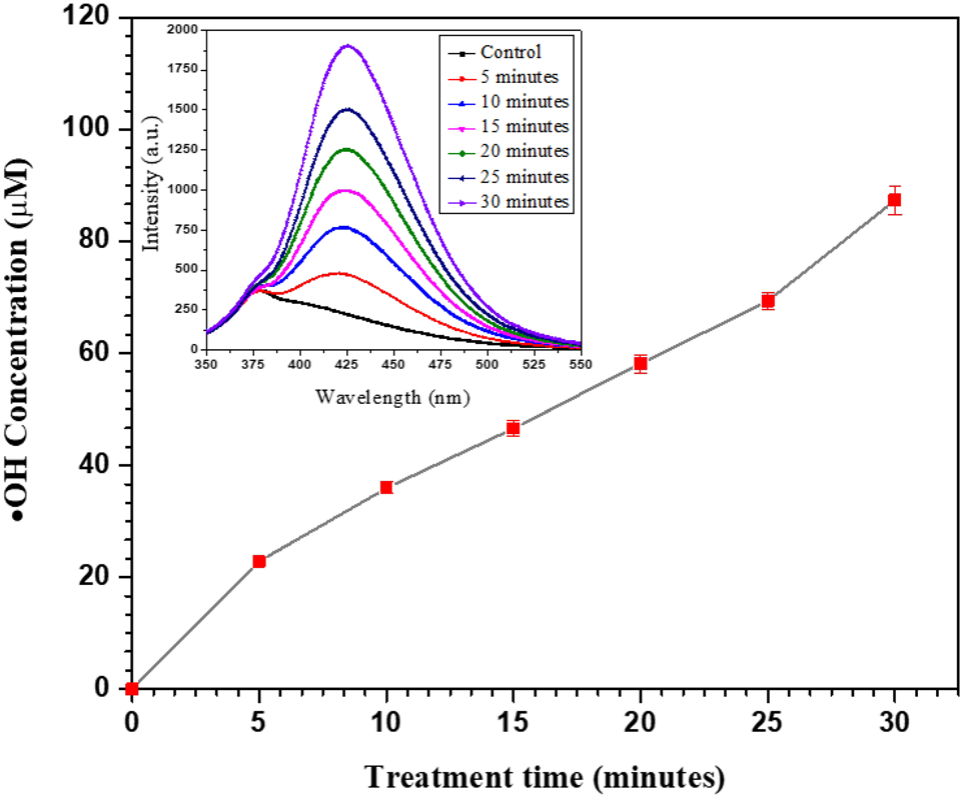
The fluorescence spectra of the hTPA solutions (inset) and the time-varying concentrations of $\cdot {\text{OH}}$ generated by the developed device at 4 kV/20 kHz.

For the quantification of $\cdot {\text{OH}}$, we have utilized a terephthalic probe^59,60^. An inset in Fig. 7 shows the fluorescence spectra of aqueous terephthalic acid (TPA) solutions exposed to the unit to confirm that the developed device is generating $\cdot {\text{OH}}$ in the environment. An aqueous solution of TPA is prepared by dissolving it in distilled water containing sodium hydroxide (NaOH). The initial concentrations of TPA and NaOH are 2 mM and 5 mM, respectively. The performance of the device is analyzed for different exposure times, i.e., 5 min, 10 min, 15 min, 20 min, 25 min, and 30 min. Samples of ~ 5 mL treated TPA solutions are analyzed within three hours of performing the experiments through a fluorescence spectrophotometer. The excitation wavelength is set at 315 nm, and the fluorescence spectra are collected for these times in the spectral range between 350 and 550 nm at an emission wavelength of 425 nm. The TPA can be oxidized into hTPA by $\cdot {\text{OH}}$ in an aqueous solution, which emits light at 425 nm (see inset in Fig. 7). The peak for these samples has shown consistent enhancement in the intensity at the emission wavelength that confirms the continuous generation of $\cdot {\text{OH}}$ from the device.

This Fig. 7 further shows the time-dependent variation in the concentration of $\cdot {\text{OH}}$ in the hTPA solution. By using the known concentration of hTPA, a calibration curve is obtained, and from the fluorescence spectra, the concentration of hTPA can be calculated. When the treatment time varied from 5 to 30 min, the concentration of $\cdot {\text{OH}}$ significantly increased, almost linearly, from 22.78 to 87.24 µM.

Results suggest that the device is producing positively and negatively charged ions along with $\cdot {\text{OH}}$ which are utilized to eliminate different airborne and pathogenic microorganisms. The lipid peroxidation can produce transient pores through cold-plasma detergent ions for wall rupture of bacteria/viruses in the indoor environment. Furthermore, since the plasma discharge happens in the air, in balancing positive and negative ions, the electron pulsation will receive a certain amount of energy. When the electrons collide with the bacteria and the mold scorpion, the energy transmitted will be similar to the bond energy of the chemical bond, the bond will be broken and bacteria/mold/fungus may no longer breed^61^.

**Figure 8:**
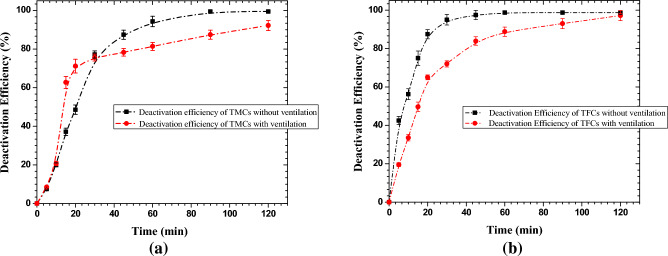
The deactivation efficiencies of (**a**) total microbial counts (TMCs) and (**b**) total fungal counts (TFCs) in with and without ventilation cases.

To investigate the elimination of TMCs and TFCs by the developed cold-plasma detergent in environment device, the air purifying unit was operated at the optimized parameters in an indoor space measuring 3 × 2.4 × 2.4 m^3^ with and without ventilation. Air sampling is carried out with the air sampler before operating the device and then after 15 min, 30 min, 45 min, 60 min, 90 min, and 120 min of continuous operation. The control of TMCs was 175 CFU/m^3^ and 295 CFU/m^3^, respectively from the indoor environment in the test room for both the cases of with and without ventilation. Figure 8a illustrates the deactivation efficiency of TMCs at different time intervals in both cases. In the case of without ventilation, more than 99% deactivation efficiency has been achieved in 90 min of continuous operation of the source, while more than 94% deactivation efficiency has been achieved in 60 min of continuous operation of the source. In the case of with ventilation, the deactivation efficiency dropped slightly. Around 92% deactivation efficiency has been achieved in around 120 min.

**Figure 9:**
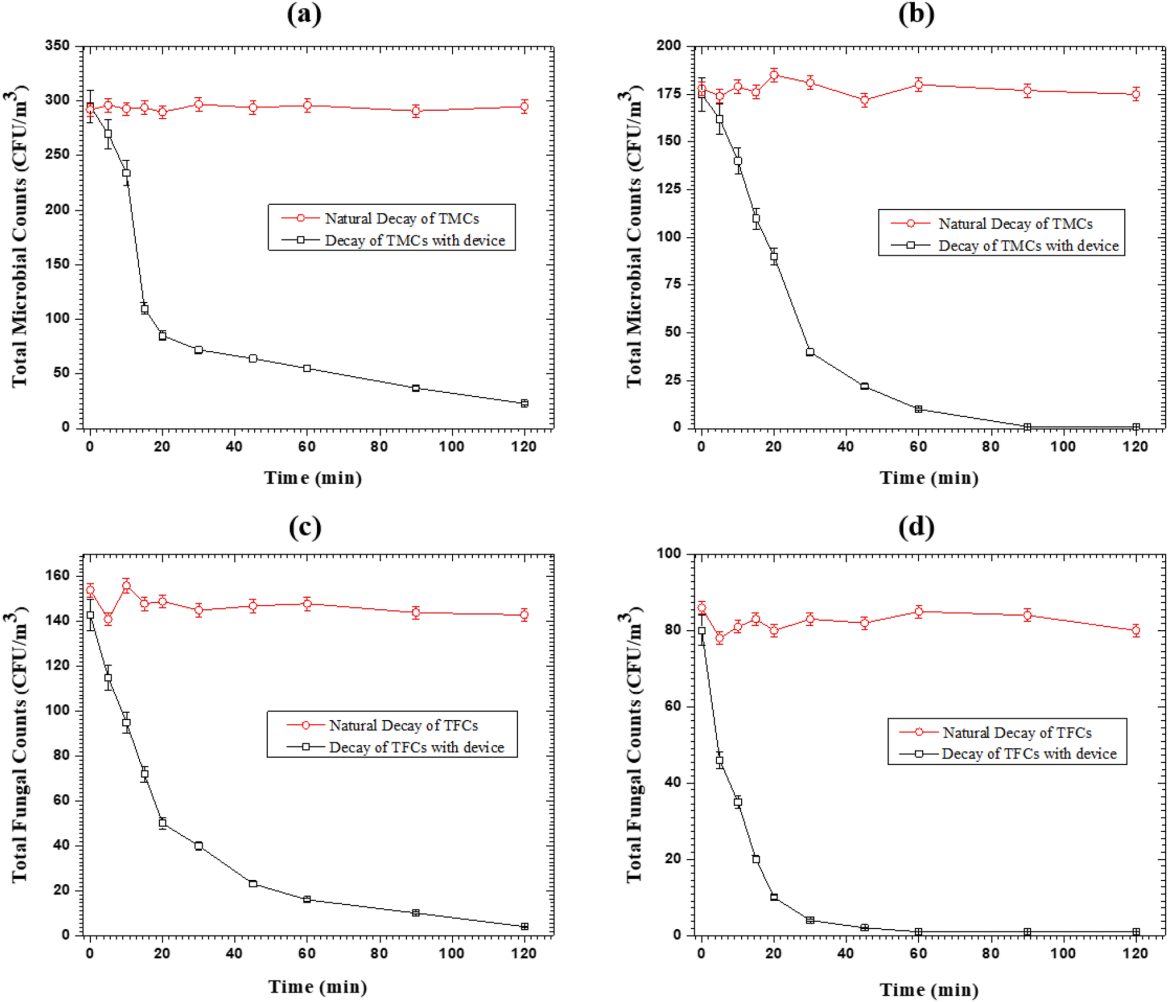
Decay of the counts of TMCs and TFCs naturally and with the operation of cold plasma detergent device (**a**, **c**) in the case of ventilation, (**b**, **d**) in the case of without ventilation.

Figure 8b shows the deactivation efficiency of TFCs at different time intervals in both cases. The controls of TFCs were around 80 CFU/m^3^ and 143 CFU/m^3^, respectively, for both with and without ventilation cases. In continuous 30 min of operation of the device, more than 95% deactivation efficiency has been achieved, and more than 99% deactivation efficiency has been achieved in 60 min of continuous operation for the case of without ventilation. On the other hand, with ventilation, the deactivation efficiency has been around 72% and 89% in continuous 30 min and 60 min of operation of the source, respectively. Tests have also been performed to see the natural decay of the counts of TMCs and TFCs in both cases, with and without ventilation. The comparative results of their decay along with natural decay are shown in Fig. 9 and no significant effect of natural decay has been observed in the tested environment. A similar study in a closed chamber has been carried out by Comini et al. (2021) and have shown that by positive and negative air ions, more than 70% inactivation of *E.coli* took about 8 h^62^.

**Figure 10:**
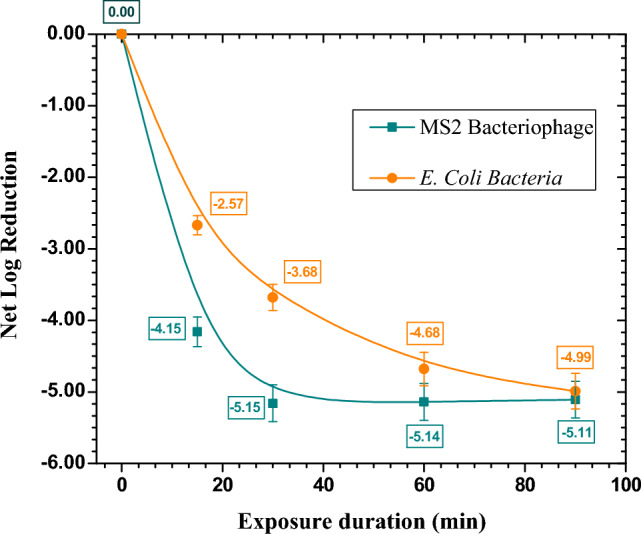
Net log reduction in case of aerosolized MS2 phage and *E. coli* bacteria.

The device efficiency in the reduction of aerosolized *E. coli* bacteria and MS2 phage in an enclosed environment measuring size 0.6 × 0.6 × 0.6 m^3^ is also tested. These aerosolized microorganisms were selected specifically for their ability to gauge the efficacy of the developed source for reducing a commonly encountered organism, such as the influenza virus and SARS-CoV-2. The net log reduction and net percentage reduction data are shown in Fig. 10 and Table 1, respectively.

**Table 1:** Deactivation effectiveness of aerosolized MS2 phage and *E. coli.*

Bioaerosol type	Species	Data type	15 min	30 min	60 min	90 min
Virus	MS2 phage	Initial Log (Control)	5.14 ± 0.25	5.14 ± 0.26	5.13 ± 0.24	5.11 ± 0.24
		Net Log Reduction	4.15 ± 0.21	5.14 ± 0.26	5.13 ± 0.26	5.11 ± 0.25
		Net % Reduction	99.993	99.999	99.999	99.999
Bacteria	*E. coli*	Initial Log (Control)	4.98 ± 0.26	4.98 ± 0.24	4.97 ± 0.25	4.99 ± 0.25
		Net Log Reduction	2.57 ± 0.13	3.68 ± 0.18	4.68 ± 0.23	4.99 ± 0.25
		Net % Reduction	99.733	99.979	99.998	99.999

When tested against MS2 phage, the device showed a maximum of 99.999% reduction in 30 min while reaching a 99.99% reduction, it took only 15 min. When tested against *E. coli*, the developed device has shown a maximum reduction of 99.999% in 90 min while reaching a 99.98% reduction, it took 30 min. In both cases, the initial reduction is very fast, more than 99% in 15 min of continuation operation of the air purifying unit. In this study, the phenomenal performance of the airborne microorganism’s elimination has been accomplished through the combination of negative and positive ions with the hydroxyl radicals.

The decrease in the number of CFUs and PFUs is mostly caused by the cold-plasma detergent as the exposure time increased. Especially for the *E. coli* bacteria, the reduction in CFUs is comparatively lower with increasing treatment time because of their self-defense and self-healing mechanisms that protect them^63,64^. In contrast, when the plasma exposure time is longer, a higher number of cold-plasma detergent species are generated, causing damage to the outer cell membrane of *E. coli* bacteria, which immediately suppresses the self-healing protection mechanism of the *E. coli* bacteria, resulting in a higher deactivation efficiency. In general, there are multiple repair systems in bacteria, i.e., *E. coli,* such as lipid remodeling (one of the outer membrane components), membrane repair proteins such as MreB and MreD, and enzymes such as catalase, superoxide dismutase (neutralize the ROS), and autolysins. Besides these, there is a stress response that can activate the gene series. These genes help activate sigma factors such as sigmaB and sigmaE. All these factors repairs the damaged cell wall. Self-defence in *E. coli* includes bacteriocins (kills other bacteria), efflux pump (expel antibiotics out from the cell), sigma factor RpoS (activate during stress), quorum sensing, and genetic changes such as gene transfer (for acquiring the antibiotic resistance)^65,66^.

Table 2 shows a comparison of the log reduction of MS2 phage with different NTP reactor configurations known in the literature. As quite evident, the log reduction of MS2 phage in this study is higher than that of the earlier known works using different NTP configurations. The time taken by the system to achieve maximum log reduction is also lower than that using different NTP reactor configurations. In fact, the complete deactivation of any virus depends on different properties, like its genome type (i.e., ssRNA or dsRNA), enveloped or non-enveloped, and its genome size. If we compare the different characteristics of SARS-CoV-2 and MS2 Bacteriophage, then the genome size of SARS-CoV-2 is 29.8 kb (enveloped), while the genome size of MS2 Bacteriophage is 3.6 kb (non-enveloped). Viruses with larger genomes generally inactivate more quickly because their larger size makes them more susceptible to hydroxyl radical-based damage. Due to its lower genome size and non-enveloped characteristics, it is a bit difficult to deactivate MS2 phage in comparison to the SARS-CoV-2^67^.

**Table 2:** A comparison of log reduction of MS2 bacteriophage with different NTP reactor configurations.

Sr. No	Virus	NTP reactor configuration	Initial concentration	Log reduction	Time (Minutes)	References
1	MS-2 bacteriophage	Packed-Bed Reactor	10^6^ pfu/ml	2.35	45	^46^
2	MS-2 bacteriophage (on FFRs)	SDBD + Water Mist	10^6^–10^8^ pfu/piece	2.00	20	^68^
3	MS-2 bacteriophage	Corona Discharge	10^10^ pfu/ml	4.00	NA	^69^
4	MS-2 bacteriophage	PCO + HEPA Filter	10^10^ pfu/ml	4.00	35	^70^
5	MS-2 bacteriophage	Bi-Polar Ionization & PCO	10^5^ pfu/ml	0.88 & 1.80	60	^71^
6	MS-2 bacteriophage	SDBD (Cold-Plasma Detergent)	> 10^5^ pfu/ml	> 5.00	30	Present work

This comparison shows the effectiveness of the developed device. The scope of this device is not limited only in the elimination of total microbial counts, total fungal counts, but it can also deactivate the most lethal range of other airborne bacteria and viruses perhaps including SARS-CoV-2, SARS CoV, Influenza, etc. It is because the virus is not a living organism, but a protein molecule covered by a protective layer of lipid (fat) and the produced active plasma detergent ions for more than 25 s with optimum concentration can deactivate such viruses. It is a well-known fact that any soap or detergent is the best remedy for Influenza viruses from hands because the foam cuts the fat. One needs to rub so much, for ~ 20 s or more, to make a lot of foam. By dissolving the fat layer, the protein molecule disperses and breaks on its own. It is expected that the generated cold plasma detergent with sufficient average sustenance time of active negative ions in the environment can work well for the long living pathogens, such as SARS-CoV-2, SARS-CoV, Influenza, etc.

## Conclusion

In summary, we have developed a portable cold-plasma detergent in environment device for efficient generation of active negative and positive air ions along with hydroxyl radicals similar to mother nature in a coaxial DBD configuration. The device is able to generate efficient plasma detergent ions in the indoor environment without any additional gas and works at atmospheric pressure. A 100 mm device is able to produce active ions with concentrations varying from 300 to 5,00,000 ions/cm^3^ and above. It is also able to generate negative ions with an average sustenance time of more than 25 s. The device is easily scalable in length, width and ion concentrations. The average consumed power per unit arc length by this device is found to be ~ 0.33 W/cm. The device is quite effective against aerosolized pathogens and is capable of reduction of total microbial counts and total fungal counts by more than 99%, *E. coli* bacteria by more than 99.999% and RNA-based virus *-*MS2 phage by more than 99.999% in a shorter operation time (< 90 min) of the air purifier unit in the indoor environments tested in sizes up to 3 × 2.4 × 2.4 m^3^. The synergistic effect of both air ions along with the generated hydroxyl radicals are possibly accountable for the obtained higher disinfection efficiency.

## Data Availability

The datasets generated during and/or analyzed during the current study are available from the corresponding author on reasonable request.
